# A forward-backward fragment assembling algorithm for the identification of genomic amplification and deletion breakpoints using high-density single nucleotide polymorphism (SNP) array

**DOI:** 10.1186/1471-2105-8-145

**Published:** 2007-05-03

**Authors:** Tianwei Yu, Hui Ye, Wei Sun, Ker-Chau Li, Zugen Chen, Sharoni Jacobs, Dione K Bailey, David T Wong, Xiaofeng Zhou

**Affiliations:** 1 Department of Biostatistics, Rollins School of Public Health, Emory University, Atlanta, GA, USA; 2 Center for Molecular Biology of Oral Diseases, College of Dentistry, University of Illinois at Chicago, Chicago, IL, USA; 3 Shanghai Children's Medical Center, Shanghai Jiao-Tong University, Shanghai, China; 4 Department of Statistics, University of California at Los Angeles, CA, USA; 5 Department of Human Genetics & Microarray Core, University of California at Los Angeles, Los Angeles, CA, USA; 6 Affymetrix, Inc., 3420 Central Expressway, Santa Clara, CA, USA; 7 Dental Research Institute, School of Dentistry, David Geffen School of Medicine & Henry Samueli School of Engineering & Jonsson Comprehensive Cancer Center, University of California at Los Angeles, Los Angeles, CA, USA; 8 Guanghua School & Research Institute of Stomatology, Sun Yat-Sen University, Guangzhou, China

## Abstract

**Background:**

DNA copy number aberration (CNA) is one of the key characteristics of cancer cells. Recent studies demonstrated the feasibility of utilizing high density single nucleotide polymorphism (SNP) genotyping arrays to detect CNA. Compared with the two-color array-based comparative genomic hybridization (array-CGH), the SNP arrays offer much higher probe density and lower signal-to-noise ratio at the single SNP level. To accurately identify small segments of CNA from SNP array data, segmentation methods that are sensitive to CNA while resistant to noise are required.

**Results:**

We have developed a highly sensitive algorithm for the edge detection of copy number data which is especially suitable for the SNP array-based copy number data. The method consists of an over-sensitive edge-detection step and a test-based forward-backward edge selection step.

**Conclusion:**

Using simulations constructed from real experimental data, the method shows high sensitivity and specificity in detecting small copy number changes in focused regions. The method is implemented in an R package FASeg, which includes data processing and visualization utilities, as well as libraries for processing Affymetrix SNP array data.

## Background

Most human cancers are characterized by genomic instabilities. In-depth knowledge of genomic aberrations has important clinical values in diagnosis, treatment, and prognostics of cancer [1]. Genomic aberrations can be analyzed using a variety of high-throughput genetic and molecular technologies, such as array-based comparative genomic hybridization (array-CGH) [2] and SNP array-based copy number analysis [3]. A number of methods have been developed to perform smoothing and/or to detect edges of segments containing one consistent copy number [4-23], some of which were compared and summarized by Lai *et al*. and Willenbrock *et al*. [24,25].

High-density array platforms, e.g. SNP array, provide the opportunity to identify genomic aberrations that localize to small segments of the chromosome, which we refer to as focused CNA in this paper. To analyze the DNA copy number of a disease sample, the matched normal DNA can be used as a reference for the computation. While this approach yields relatively low noise, such a matched normal DNA sample is often unavailable. By using the existing SNP array data libraries derived from large numbers of normal samples, disease samples can now be analyzed without paired normal samples [14,26]. However, proper handling of the data is necessary to lower the noise and avoid identifying large numbers of false-positive CNA segments. One way to achieve this goal is to reduce noise at the probe level, by selecting probes based on dose response to copy number change [26] or sequence properties [27]. Another approach is to apply data smoothing and segmentation methods with high sensitivity and specificity. While most methods designed for array-CGH data can potentially be applied, their parameters may need to be fine-tuned to adapt to the different characteristics of the SNP array data. Here we present a test-based data segmentation method. In our algorithm, each chromosome is first broken into small segments through an over-sensitive edge detection mechanism. The consecutive segments are then iteratively merged by local testing, using a forward-backward edge selection scheme, until all remaining edges pass a significance threshold. The data sets used in this study were generated with Affymetrix GeneChip^® ^Mapping 50 K Xba arrays on two model cell lines with known genomic alterations and two tumor DNA samples of oral squamous cell carcinoma.

## Results and discussions

The SNP array results on two model cell lines were generated as described in the Methods section for the development and testing of our algorithm. The cell lines used here were GM03226 with a known trisomic aberration segment in chromosome 9 [9pter > q11], and GM00870 with a known single copy deletion segment in chromosome 9 [9pter > p21]. The data was first processed with Copy Number Analysis Tool (CNAT 3.0) from Affymetrix Inc, which utilizes Huang *et al*.'s method to estimate SNP-level copy numbers based on libraries of normal samples [14]. We chose CNAT because of its widespread use for the analysis of SNP array data. Better data pre-processing methods [26,27] may lead to better results than reported here. Following the CNAT process, the SNP-level copy number values were log_2 _transformed to achieve near-normal distributed copy numbers. The mean and standard deviation (SD) for the signals from one, two and three copies were defined based on knowledge of the cell lines. We found that normal two-copy DNA yielded a mean of 1.03 and SD of 0.77; single-copy DNA yielded a mean of 0.25 and SD of 0.63; and three-copy DNA yielded a mean of 1.45 and SD of 0.91. Compared to single-copy DNA, three-copy DNA has a mean that is closer to two-copy DNA, and a larger standard deviation. Hence three-copy aberrations are harder to detect than single-copy aberrations.

**Figure 1:**
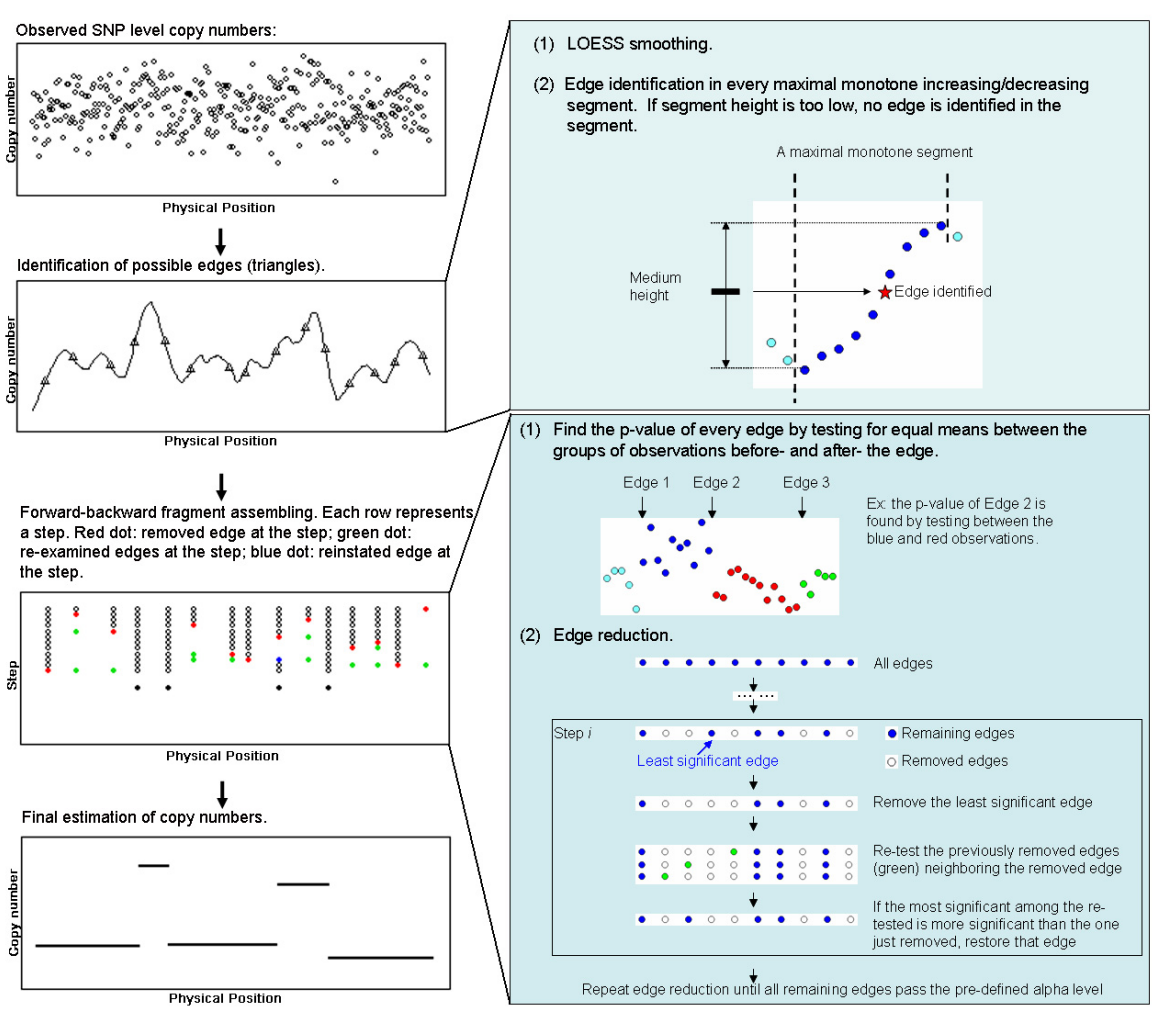
An illustration of the workflow of the forward-backward fragment assembling (FASeg) method.

Simulated chromosomes with focused CNA were constructed based on the SNP array results on model cell lines (GM03226 and GM00870) as described in the Methods section. An algorithm which is effective in identifying focused copy number aberrations was developed and tested based on these simulated chromosomes. In the following text, we refer to the algorithm as FASeg (fragment assembling segmentation), which is also the name of the R package. The work flow is illustrated in Figure 1. The optimal parameters for focused CNA detection were determined based on the simulated chromosomes. Preliminary testing was also performed using the simulated chromosomes.

**Figure 2:**
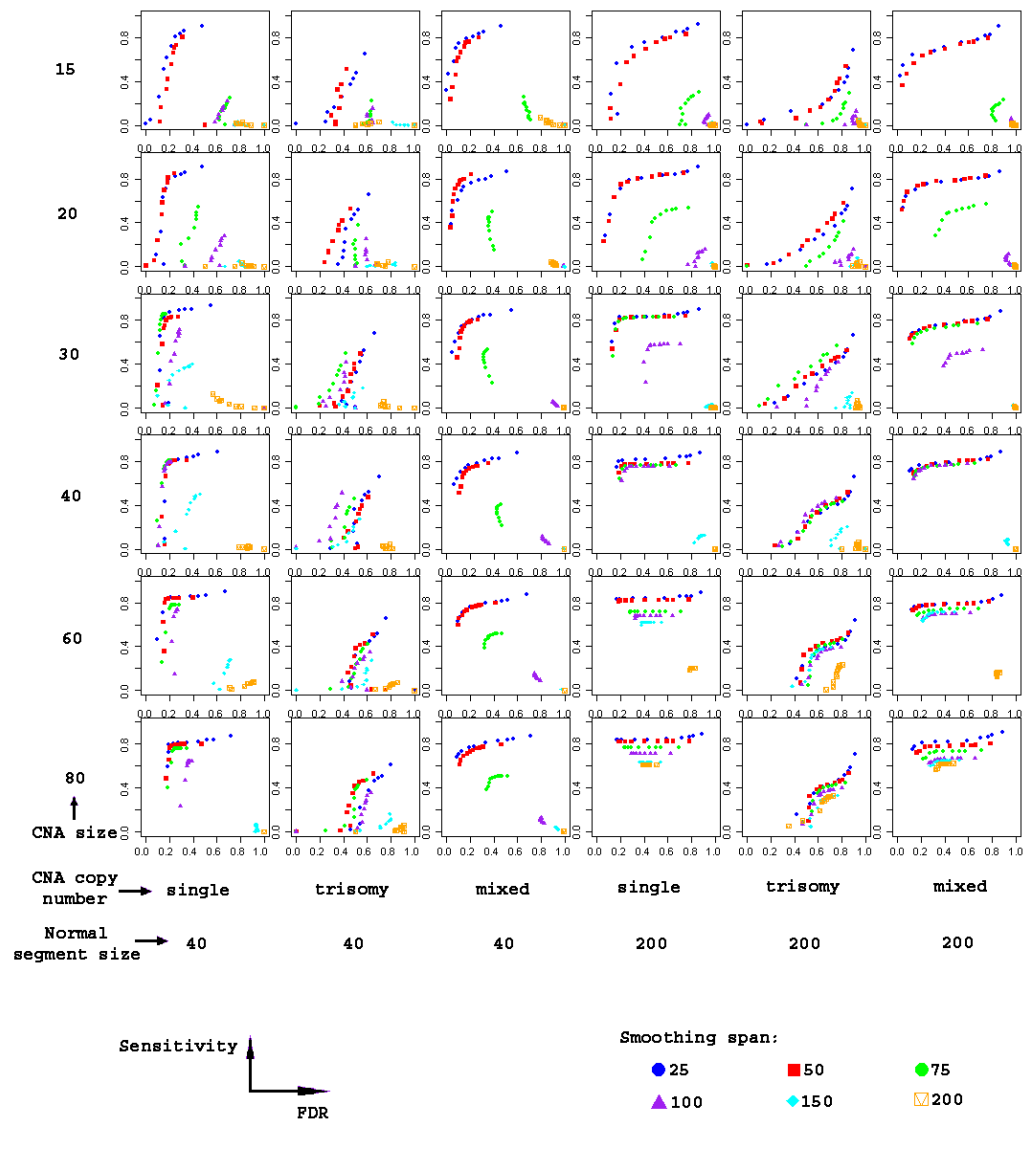
The effect of smoothing span on the sensitivity to detect CNA segments. Every sub-plot is based on 100 simulated chromosomes, each harboring 6 normal segments and 5 CNA segments. Ten alpha levels were examined at each smoothing span.

There are two major parameters that influence the results of edge identification. One is the span of the initial smoothing. The other is the p-value cutoff to define the significance of each edge. In our algorithm, the smoothing span is expressed in terms of number of SNPs. After dividing by the total number of SNPs in a chromosome, it is transformed into the α value in the LOESS smoothing of the data, which controls the degree of smoothing. Six span values, 25, 50, 75, 100, 150, and 200 were tested in simulations using a range of p-value cutoffs (Table 1). We tested 36 different combinations of CNA segment size, CNA copy number and normal segment size using simulation. For each combination, 100 simulated chromosomes were analyzed. Because of the high noise level in the data, we allowed a tolerance distance of 5 SNPs when matching true edges with identified edges. For each simulation setting, the sensitivity was plotted against the false-discovery rate (FDR) to show the performance (Figure 2). We found that the smoothing span values of 25 and 50 performed similarly in most cases. The smoothing span of 25 was chosen as the default value.

**Table 1:** Parameters tested for the seven R packages

Packages		Parameters tested		
Tuned packages		Tuning parameter	Values tested	Other parameters
	
	FASeg	Sig	0.25, 0.1, 0.075, 0.05, 0.025, 0.01, 0.005, 0.001, 0.0001, 0.00001	Default
	aCGH	Vr	10, 7, 5, 2, 1, 0.5, 0.1, 0.05, 0.01, 0.001	Default
	DNAcopy	alpha	0.25, 0.1, 0.075, 0.05, 0.025, 0.01, 0.005, 0.001,0.0005, 0.0001	* nperm = 1000
	GLAD	qlambda	0.75, 0.9, 0.925, 0.95, 0.975, 0.99, 0.9925, 0.995, 0.9975, 0.999	** lambdabreak = 0.01 lambdacluster = 0.01 lambdaclusterGen = 0.01 param = c(d = 1)
Packages examined at default setting	Picard	Maxk = max(true segment size) + 5, maxSeg= #(true segments) + 1		
	RJaCGH	*** burnin = 50, *** TOT = 500, jump.parameters = NULL, k.max = #(true states) + 1		
	BioHMM	Default		

* The change in the number of permutations is to reduce computing time. Experiments showed that reducing the number from 10000 to 1000 has minimal effect on the outcome.

** These parameters were tuned according to the GLAD manual to increase sensitivity. Using default values, the method detected limited number of edges from noisy data.

*** The purpose of reducing the number of iterations was to save computing time.

**Figure 3:**
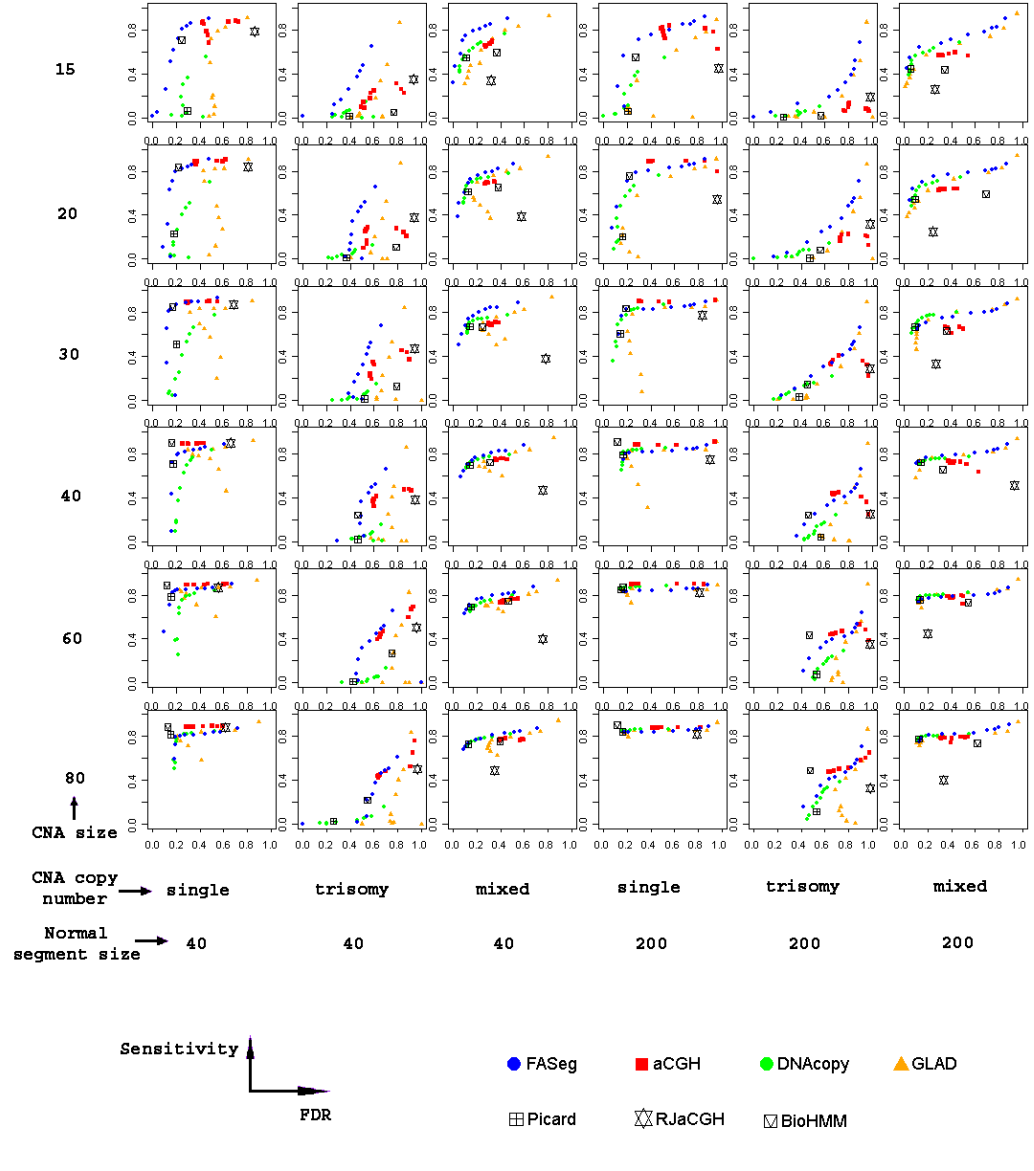
The comparison of the performance of seven methodsavailable as R packages. Every sub-plot is based on 100 simulated chromosomes, each harboring 6 normal segments and 5 CNA segments. FASeg, aCGH, DNAcopy and GLAD were each run at 10 parameter settings; Picard, RJaCGH and BioHMM were run at default settings. The parameters used are detailed in Table 1.

To evaluate the performance of FASeg in the context of existing methods, we ran the simulation together with six other methods (Table 1), all of which are implemented in R packages. Three of the methods could be easily tuned to change the sensitivity. They include the Hidden-Makov Model-based method in the aCGH package [23], the Circular Binary Segmentation method in the DNAcopy package [10], and the Gain and Loss Analysis of DNA method in the GLAD package [15]. For each of these packages, we identified the most influential parameter and tested 10 values around the default of the parameter in an attempt to improve its performance on noisy data (Table 1). Three other packages do not have obvious tuning parameters. They include two Hidden Markov Model-based methods in the RJaCGH package [21] and the snapCGH package [20] (referred to as BioHMM), and the dynamic programming-based method by Picard *et al*. [11] (referred to as Picard) which was run through a wrapper function in the snapCGH package. A total of 36 different combinations of CNA segment size, CNA copy number and normal segment size were tested. We compared the performance of the seven packages by plotting the sensitivity against FDR (Figure 3). We noticed that most of the methods tested here did not show the typical monotone ROC type of curve. The results indicated that FASeg was particularly sensitive to CNA segments that were small in size and low in signal-to-noise ratio (three copies, columns 2 and 5 in Figure 3). GLAD showed similar performance when the flanking normal segments were relatively long (200 SNP markers, column 5 in Figure 3). BioHMM and aCGH performed best when the signal-to-noise ratio was low while the CNA segments and the flanking normal segments were long (column 5 in Figure 3). For situations with relatively higher signal-to-noise ratio (single copy, columns 1 and 4 in Figure 3), most of the methods tested here performed reasonably well, with BioHMM and FASeg leading the performance when CNA segments were relatively small (40 SNP markers or less). With chromosomes that contained multiple CNA segments at different copy numbers (columns 3 and 6 in Figure 3), FASeg, DNAcopy, GLAD and Picard achieved the best performance. One observation is that HMM-based methods tend to be less effective when multiple CNA states were present in a single chromosome. Again we allowed a tolerance distance of 5 SNPs when matching true edges with identified edges. Similar results were obtained when other tolerance values (3 and 7) were used [see Additional file 1]. Additional analyses were performed using the default setting of each package [see Additional file 1]. Because some of the methods tested here were tuned to adapt to data with higher signal-to-noise ratio, the results at default settings may not be representative of the performance shown in Figure 3.

**Figure 4:**
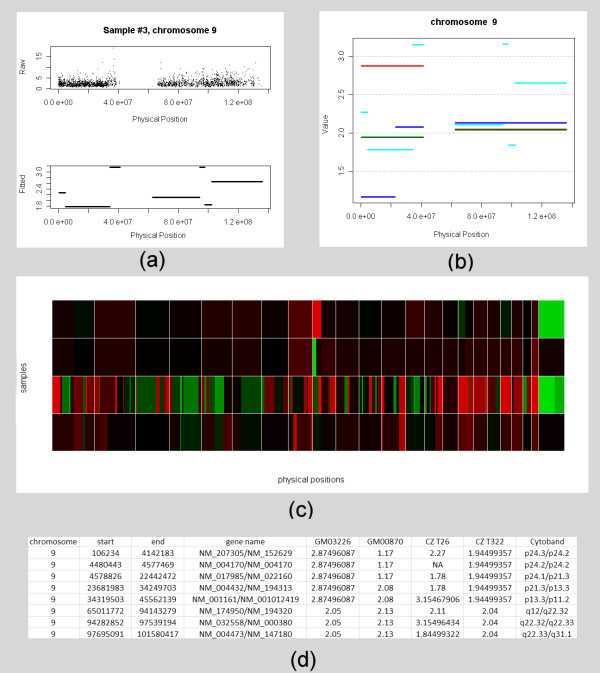
Sample output of the R-package FASeg. The results were obtained using the smoothing span of 50 SNPs and the alpha level of 10^-6^. (a) Raw copy number (upper panel) and fitted values (lower panel) of chromosome 9 for data from the Mapping 50 K Xba array, generated from an oral squamous cell carcinoma case (CZ T26). (b) Comparison of the copy numbers for chromosome 9 between four samples. Two primary skin fibroblast cell lines: GM03226 (with a known trisomic segment in chromosome 9 [9pter > q11]; red) and GM00870 (with a known single copy deletion segment in chromosome 9 [9pter > p21]; blue). Two previously uncharacterized oral squamous cell carcinoma cases: CZ T26 (green) and CZ T322 (aqua). (c) Color display of the fitted values of the whole genome for all four samples. From top to bottom: GM03226, GM00870, CZ T26 and CZ T322. The gridlines separate chromosomes lined up in numerical order, with the X chromosome being the last. Black: normal; red: higher; green: lower. (d) A section of the condensed table output containing copy number and Cytoband information for samples GM03226, GM00870, CZ T26, and CZ T322.

An R package, named FASeg, was developed to implement the algorithm. In addition to the core algorithm described above, this package also consists of data pre-processing, visualization and gene-level data summarizing functions. The core function accepts simple matrix input and produces simple matrix output, which makes it easily adaptable for data input from various platforms. The package is available at the FASeg website [28]. Plain-text probe level data exported from Affymetrix GTYPE/GDAS can be read into R in batch mode and converted to a single matrix of raw copy numbers, using a simplified version of the method by Huang *et al*. [14]. For the Mapping 500 K arrays, currently this is the only mechanism for data input. For the Mapping 100 K array data, plain-text output of raw copy numbers from CNAT can be loaded as an alternative. The raw copy number matrix, which can include multiple array data sets, is processed by the core algorithm to produce segment-wise constant estimates. The expected measurement value corresponding to two copies can be input, which will induce the function to perform further re-scaling of the fitted data based on cluster analysis. The fitted data can be displayed in a few modes: single experiment with raw and fitted values side-by-side (Figure 4A); multiple experiments/single chromosome (Figure 4B); multiple experiments/all chromosomes (Figure 4C). Portions of the data can be plotted by simple matrix manipulation. From the fitted data, gene-level copy number and Cytoband information [29] can be summarized in a table. The table can be collapsed by merging nearby genes that show the same copy numbers in all experiments (Figure 4D).

The computing speed of the FASeg package was tested against the existing packages listed in Table 1 using the complex cancer sample CZ T26. The average computing time over all parameter settings are reported in Table 2. DNAcopy exhibited the fastest computing speed among the packages tested. GLAD, Picard and aCGH showed similar computing speed, at around 1.7 minutes per sample. FASeg used about 3 minutes. From Figure 2, we observed that FASeg showed similar performance when the smoothing span is 50, as compared to the default value of 25. When the smoothing span of 50 was used, FASeg achieved 1.4 minutes in the speed test. BioHMM and RJaCGH were far behind in terms of computing speed. In the simulations, we noticed that RJaCGH could be much faster when the jump parameters were given.

**Table 2:** Comparison of computing time*

	CPU time (seconds)
FASeg	181
aCGH	107
DNAcopy	18
GLAD	98
Picard	101
RJaCGH	13778
BioHMM	1619

* Comparison was made in R 2.4.1 on a desktop computer running the Windows XP^® ^operating system. CPU: AMD Athlon 64 3800+ @ 2.4 GHz; RAM: 1.2 Gb. The CPU time for the tumor sample CZ T26 was reported. For FASeg, aCGH, DNAcopy and GLAD, the ten parameters listed in Table 1 were tested and the average CPU time was reported. For Picard, ten maxSeg values between 2 and 20 were tested and the average CPU time was reported. For RJaCGH and BioHMM, the parameters listed in Table 1 were used.

**Figure 5:**
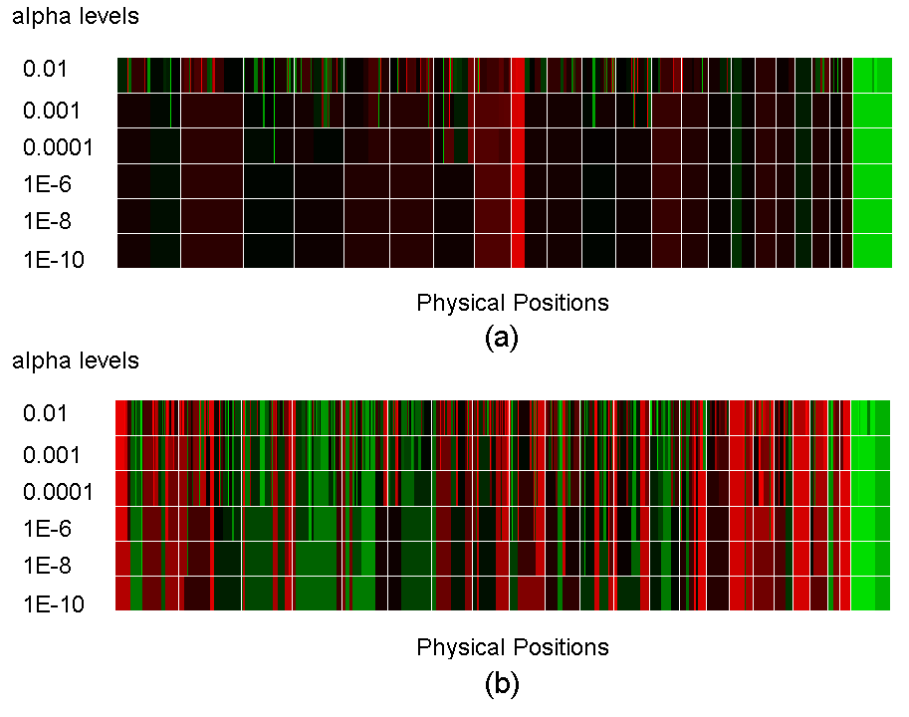
Demonstration of the performance of FASeg at different p-value cutoffs. Fitted values at each p-value cutoff were displayed on the left. The gridlines separate chromosomes lined up in numerical order, with the X chromosome being the last. Black: normal; red: higher; green: lower. (a) GM03226 cell line data; (b) CZ T26 cancer tissue data.

There is a trade-off between sensitivity and specificity in edge detection. We illustrate the behavior of FASeg in Figure 5, where we applied multiple p-value cutoffs to the model cell line GM03226 and the oral cancer sample CZ T26. Results from the other two samples were similar (data not shown). With the cell line GM03226, which only has a trisomic segment in chromosome 9, we saw that larger p-value cutoffs yielded some false CNA segments that were mostly small in size, while the true trisomic segment was consistently identified (Fig. 5a). With the CZ T26 cancer tissue sample, we observed many small segments when less stringent cutoffs were used (Fig. 5b), while we were unable to judge the validity of the segments. The edges identified using a smaller p-value cutoff were mostly in a subset of the edges identified using a larger p-value cutoff (Fig. 5). When tuning the p-value cutoff parameter, the user can visually examine the raw data around the edges that disappear when the cutoff level is lowered, and decide whether the change of cutoff value is reasonable.

When the intent is not finding focused CNA, or there is a strong prior belief that the CNA segments are not focused, more stringent p-value cutoffs should be used. On the other hand, if the intent is to identify focused CNA, less stringent cutoffs have to be used with the risk of identifying false CNA segments. However, the problem of finding false-positive segments is not as severe as it seems in most applications, where multiple samples are analyzed to identify CNAs over-represented in a subgroup of samples. In such applications, after data segmentation, cross-sample testing is performed to find CNA segments consistent across many samples. False segments at random locations will most likely be inconsistent across samples, hence insignificant in the cross-sample testing.

## Conclusion

In summary, we presented an algorithm to find break points in copy number data. It consists of an over-sensitive edge detection step and a test-based segment merging step. After the over-sensitive edge detection step, the segmentation task becomes a model-selection task. In the forward-backward model selection, by using the common segment-wise Gaussian assumption, the backward step is reduced to a manageable local search. The model makes no assumption about the number of CNA states in a chromosome. Thus it provides the flexibility to handle multiple CNA states in a single chromosome, which is important in the analysis of complex cancer samples. In the implementation of this algorithm into a user-friendly R package (FASeg), we optimized the parameters for identifying focused CNA in noisy data. In simulation studies based on real data, FASeg was sensitive to CNA segments that were small in size and low in signal-to-noise ratio. It performed well when presented with complex samples with multiple CNA states per chromosome. From the users' stand point, FASeg is intuitive and easy to tune.

## Methods

### The forward-backward fragment-assembling algorithm

The array-based copy number data consists of a series of *N *observations *{*(*X*_1_, *Y*_1_), ..., (*X*_*N*_, *Y*_*N*_)*} *for each chromosome, in which *X*_*i*_'s are positions along the chromosome and *Y*_*i*_'s are log_2_-ratios in the aCGH data or processed copy numbers from SNP array data. Between any twoconsecutive edges, which remain to be identified, we assume a local constant model with Gaussian error. We apply a two step method for the identification of edges. The first step aims to identify most true edges, at the price of identifying possible false-positive edges. This step is a high sensitivity and low specificity step. In the second step, the goal is to remove the false-positive edges, while retaining the true edges through statistical model selection. Figure 1 shows an illustration of the workflow.

#### Step 1. Over-sensitive edge detection

To identify all possible edges, we apply an *ad hoc *method, which is based on one-dimensional differential edge detection. At this step, the actual *X *values are ignored, because the main interest is to find copy number changes between data points. (1) To reduce noise, a locally weighted regression smoother (LOESS) with Gaussian kernel is fitted through the data *Y*_1_,....., *Y*_*N *_to generate fitted values *Y*_1_',....., *Y*_*N*_'. (2) An edge is identified in every maximal monotone increasing/decreasing segment in the LOESS fitted curve. The edge position is assigned between the two observations that span the medium height of the segment (Figure 1). If the height of the segment is below a predefined threshold value, the edge is removed. The threshold value should be set such that copy number changes at or below that level is ignorable. The default value in the FASeg package is 0.1.

#### Step 2. Forward-backward edge reduction

After step 1, the data is overly fragmented into small pieces. The next step is to merge the fragments by statistical testing. The task amounts to a model selection problem with a large number of candidate predictors (edges). The full model is the model that contains all the edges identified in Step 1. We resort to the forward-backward scheme to quickly reduce the full model to a smaller model containing fewer edges. In the segment-wise constant model, the removal of an edge only affects the likelihood of the data points between the previous and the next edge. Thus a local ANOVA test, or unpaired t-test, is equivalent to the likelihood ratio test for model selection. Also, the removal of an edge only changes the significance of the two neighboring edges. Thus in the backward selection step, only previously removed edges within the segment confined by the two neighboring edges need to be re-examined (Figure 1, green dots).

We first define p-values for all the edges. For each edge, we consider the observations between the previous and the next edges. These observations are spatially divided into two groups by the edge of interest. The unpaired Student's t-test is performed to find the significance of the division, and the p-value is associated with the edge. Second, when the p-values for all the edges are defined, we iteratively remove edges from the least significant one. With the removal of each edge, all previously removed edges around this edge are re-examined. For example, if edge *i *is being removed, with α edges immediately before edge *i *and *b *edges immediately after edge *i *having been previously removed, then for each edge *j *∈ [*i *- *a*, *i*) ∪ (*i*, *i *+ *b*], we re-compute its p-value after the removal of edge *i*. If the lowest of the p-values is smaller than that of edge *i*, the corresponding edge is reinstated. This process is iterated until all remaining p-values are smaller than a cutoff value. This p-value threshold can be user-defined and may be fine-tuned based on each sample to get the best balance between sensitivity and specificity. After the edge identification, for the segment between two consecutive edges, the median value of *Y *is taken as the estimate.

### DNA samples and the SNP array mapping assay

SNP array data was generated on 2 model cell lines (GM03226 and GM00870) with known genomic alterations and 2 previously uncharacterized oral squamous cell carcinoma samples (CZ T26 and CZ T322). Each sample was analyzed using one array. The model cell lines were obtained from Coriell Cell Repositories/NIGMS [30]. GM03226 are fibroblasts with a trisomic segment in chromosome 9 [9pter > q11], and GM00870 are fibroblasts that are known to have a single copy deletion segment in chromosome 9 [9pter > p21]. DNA labeling, hybridization, washing and staining of the Mapping 50 K Xba arrays were performed according to the standard Gene-Chip Mapping 100 K Assay protocol (Affymetrix). The arrays were scanned using a GeneChip Scanner 3000. The scanned array images were processed with GeneChip Operating software (GCOS) 1.3. The genotype calls and intensity of the SNP probes were generated by GeneChip DNA Analysis Software (GDAS) 1.4. The probe-level intensities were further converted to SNP level intensities using CNAT 3.0.

### Simulation based on the real data

The simulation data was generated based on the model cell lines GM03226 and GM00870. We obtained pools of SNP-level copy number values for single-, two-, and three-copy DNA. By resampling from these pools, we constructed copy number readings of the simulated chromosome. Each simulated chromosome contained 11 segments. Probesets were evenly spaced. Thus we use the number of probesets to represent the segment size. Starting from the normal segment, the chromosome construction alternated between normal segments and CNA segments. Six normal segments and five CNA segments were simulated for each chromosome. Within each simulated chromosome, a single normal segment size and a single CNA segment size were used. Two normal segment sizes (40 and 200 SNPs) and six CNA segment sizes (15, 20, 30, 40, 60 and 80 SNPs) were tested. Three settings of aberration levels were tested: (1) all five CNA segments in the chromosome represented single-copy DNA, (2) all five CNA segments were three-copy, (3) the five CNA segments were a mixture of single-copy, three-copy and segments of higher-magnitude copy number changes. No real data was available for the higher-magnitude CNA. Because such segments were easier to detect, and some deviation from the truth would not severely affect the results of performance comparison, we simulated them by adding constants to the single-copy and three-copy pools. Three new pools were created. Pool L1 was created by moving the median of the single-copy pool to log_2_(0.5) to mimic copy numbers lower than one. Pools H1 and H2 were created by moving the median of the three-copy pool to log_2_(4) and log_2_(5) respectively, to mimic copy numbers higher than 3. In the simulated chromosome, the five CNA segments were drawn from the three-copy pool, the single-copy pool, H1, L1, and H2 respectively. For each of the 2 × 6 × 3 settings, 100 chromosomes were simulated.

At each simulation parameter setting, to assess the ability of the algorithm to identify CNA segments while limiting the number of false-positive edges, we plotted the sensitivity, which is the proportion of true edges identified, against FDR, which is the proportion of false edges among all identified edges. Because of the high noise level in the data, we allowed a tolerance distance when matching true edges with identified edges. The tolerance distance is defined based on the number of SNPs. If an identified edge is equal to or less than the tolerance distance away from the true edge, we considered the true edge to be correctly detected. The results reported in Figure 2 and 3 were obtained using the tolerance distance of 5 SNPs. The results from using the tolerance distances of 3 and 7 SNPs were reported in the Supplement figures 1 and 2 [see Additional file 1]. In the ideal case, the sensitivity should approach one and the FDR should approach zero.

## Abbreviations

SNP, single nucleotide polymorphism; CNA, copy number aberration; FASeg, fragment assembling segmentation; CGH, comparative genomic hybridization; aCGH, array comparative genomic hybridization; CNAT, Copy Number Analysis Tool; SD, standard deviation; GCOS, GeneChip Operating System; GDAS, GeneChip DNA Analysis Software.

## Authors' contributions

TY conceived the idea for the project, developed the statistical algorithm and the R package, and drafted the manuscript. HY and ZC performed the laboratory analyses and conducted initial statistical analyses. WS and KCL aided in interpretation of the data, and provided general statistical guidance for the study. SJ and DKB assisted the final analyses and development of the package. DW oversaw laboratory, provided general scientific guidance for the study and revised the manuscript. XZ conceived the idea for the project, oversaw the analysis, and drafted the manuscript. All authors read and approved the final manuscript.

## Supplementary Material

Additional File 1 Supplemental tables and figures. The table showing the performance of the seven R packages at default settings, and the figures showing the performance of the seven R packages using the tolerance distance 3 and 7. Click here for file
