# Supplementary material for: A forward-backward fragment assembling algorithm for the identification of genomic amplification and deletion breakpoints using high-density single nucleotide polymorphism (SNP) array
Source: BMC Bioinformatics. 2007 May 3;8:145. doi: 10.1186/1471-2105-8-145 (PMC1868765; doi:10.1186/1471-2105-8-145)
Supplement: Additional File 1 — Supplemental tables and figures. The table showing the performance of the seven R packages at default settings, and the figures showing the performance of the seven R packages using the tolerance distance 3 and 7. [file 1471-2105-8-145-S1.doc]

**Supplemental tables and figures.**

Supporting table 1. The performance of the seven R packages at default setting ( Sensitivity / 1-FDR ).

| Normal segment size | CNA copy number | CNA segment size | FASeg | aCGH | DNAcopy | GLAD | Picard | RJaCGH | BioHMM |
| --- | --- | --- | --- | --- | --- | --- | --- | --- | --- |
| 40 | 1 | 15 | 0.6 / 0.87 | 0.91 / 0.28 | 0.04 / 0.79 | 0 / NA | 0.07 / 0.76 | 0.82 / 0.15 | 0.73 / 0.79 |
|  |  | 20 | 0.78 / 0.91 | 0.95 / 0.4 | 0.16 / 0.88 | 0 / NA | 0.24 / 0.87 | 0.88 / 0.2 | 0.88 / 0.83 |
|  |  | 30 | 0.9 / 0.93 | 0.93 / 0.55 | 0.28 / 0.86 | 0 / NA | 0.55 / 0.86 | 0.91 / 0.33 | 0.9 / 0.88 |
|  |  | 40 | 0.89 / 0.88 | 0.95 / 0.66 | 0.52 / 0.82 | 0.002 / 0.4 | 0.75 / 0.87 | 0.94 / 0.36 | 0.93 / 0.87 |
|  |  | 60 | 0.9 / 0.87 | 0.94 / 0.42 | 0.73 / 0.83 | 0.001 / 1 | 0.83 / 0.9 | 0.91 / 0.47 | 0.92 / 0.92 |
|  |  | 80 | 0.9 / 0.85 | 0.93 / 0.52 | 0.83 / 0.85 | 0.001 / 0.5 | 0.86 / 0.89 | 0.91 / 0.4 | 0.94 / 0.92 |
|  | 3 | 15 | 0.21 / 0.78 | 0.23 / 0.15 | 0.01 / 0.92 | 0 / NA | 0.02 / 0.74 | 0.35 / 0.06 | 0.05 / 0.25 |
|  |  | 20 | 0.23 / 0.81 | 0.22 / 0.13 | 0.02 / 0.75 | 0 / NA | 0.01 / 0.68 | 0.38 / 0.06 | 0.12 / 0.24 |
|  |  | 30 | 0.28 / 0.71 | 0.38 / 0.1 | 0.02 / 0.71 | 0 / NA | 0.01 / 0.52 | 0.48 / 0.06 | 0.14 / 0.22 |
|  |  | 40 | 0.35 / 0.65 | 0.48 / 0.09 | 0.03 / 0.64 | 0 / NA | 0.03 / 0.61 | 0.39 / 0.05 | 0.28 / 0.62 |
|  |  | 60 | 0.4 / 0.59 | 0.7 / 0.07 | 0.01 / 0.5 | 0 / NA | 0.01 / 0.58 | 0.51 / 0.04 | 0.31 / 0.29 |
|  |  | 80 | 0.38 / 0.55 | 0.77 / 0.05 | 0.02 / 0.8 | 0 / NA | 0.03 / 0.8 | 0.5 / 0.03 | 0.26 / 0.54 |
|  | mixed | 15 | 0.65 / 0.92 | 0.72 / 0.69 | 0.59 / 0.93 | 0.2 / 1 | 0.57 / 0.93 | 0.34 / 0.68 | 0.61 / 0.66 |
|  |  | 20 | 0.77 / 0.94 | 0.76 / 0.7 | 0.69 / 0.93 | 0.23 / 0.97 | 0.64 / 0.92 | 0.41 / 0.45 | 0.69 / 0.66 |
|  |  | 30 | 0.83 / 0.95 | 0.74 / 0.65 | 0.74 / 0.9 | 0.23 / 0.99 | 0.7 / 0.9 | 0.4 / 0.23 | 0.7 / 0.79 |
|  |  | 40 | 0.8 / 0.89 | 0.8 / 0.58 | 0.76 / 0.9 | 0.24 / 0.98 | 0.73 / 0.91 | 0.51 / 0.26 | 0.76 / 0.73 |
|  |  | 60 | 0.79 / 0.82 | 0.79 / 0.47 | 0.73 / 0.88 | 0.32 / 0.91 | 0.72 / 0.89 | 0.42 / 0.25 | 0.77 / 0.56 |
|  |  | 80 | 0.8 / 0.82 | 0.82 / 0.47 | 0.77 / 0.88 | 0.34 / 0.83 | 0.76 / 0.9 | 0.54 / 0.72 | 0.79 / 0.63 |
|  |  |  |  |  |  |  |  |  |  |
| 200 | 1 | 15 | 0.72 / 0.61 | 0.65 / 0.03 | 0.11 / 0.83 | 0 / NA | 0.06 / 0.79 | 0.47 / 0.03 | 0.57 / 0.76 |
|  |  | 20 | 0.88 / 0.68 | 0.82 / 0.04 | 0.38 / 0.92 | 0 / NA | 0.21 / 0.88 | 0.57 / 0.04 | 0.81 / 0.84 |
|  |  | 30 | 0.9 / 0.68 | 0.95 / 0.05 | 0.76 / 0.88 | 0 / 1 | 0.63 / 0.9 | 0.8 / 0.17 | 0.91 / 0.88 |
|  |  | 40 | 0.91 / 0.68 | 0.94 / 0.06 | 0.87 / 0.88 | 0.01 / 1 | 0.84 / 0.89 | 0.79 / 0.11 | 0.94 / 0.92 |
|  |  | 60 | 0.92 / 0.66 | 0.94 / 0.16 | 0.92 / 0.9 | 0.12 / 0.94 | 0.91 / 0.91 | 0.87 / 0.2 | 0.94 / 0.9 |
|  |  | 80 | 0.91 / 0.65 | 0.94 / 0.19 | 0.91 / 0.89 | 0.32 / 0.93 | 0.9 / 0.9 | 0.86 / 0.22 | 0.94 / 0.92 |
|  | 3 | 15 | 0.27 / 0.34 | 0.06 / 0.02 | 0.02 / 0.82 | 0 / NA | 0.01 / 0.75 | 0.19 / 0.02 | 0.03 / 0.61 |
|  |  | 20 | 0.35 / 0.42 | 0.13 / 0.03 | 0.03 / 0.82 | 0 / NA | 0.01 / 0.58 | 0.32 / 0.02 | 0.09 / 0.46 |
|  |  | 30 | 0.42 / 0.41 | 0.23 / 0.03 | 0.08 / 0.73 | 0 / NA | 0.03 / 0.64 | 0.3 / 0.02 | 0.18 / 0.66 |
|  |  | 40 | 0.48 / 0.41 | 0.26 / 0.03 | 0.08 / 0.58 | 0 / NA | 0.05 / 0.5 | 0.26 / 0.02 | 0.29 / 0.64 |
|  |  | 60 | 0.49 / 0.4 | 0.4 / 0.03 | 0.14 / 0.54 | 0 / NA | 0.09 / 0.55 | 0.37 / 0.02 | 0.51 / 0.63 |
|  |  | 80 | 0.49 / 0.38 | 0.67 / 0.02 | 0.22 / 0.54 | 0 / NA | 0.13 / 0.55 | 0.34 / 0.02 | 0.57 / 0.61 |
|  | mixed | 15 | 0.74 / 0.63 | 0.59 / 0.49 | 0.53 / 0.96 | 0.22 / 1 | 0.46 / 0.97 | 0.28 / 0.8 | 0.46 / 0.68 |
|  |  | 20 | 0.83 / 0.68 | 0.66 / 0.56 | 0.63 / 0.91 | 0.25 / 1 | 0.55 / 0.92 | 0.26 / 0.79 | 0.61 / 0.32 |
|  |  | 30 | 0.84 / 0.65 | 0.64 / 0.63 | 0.75 / 0.93 | 0.31 / 0.99 | 0.7 / 0.95 | 0.33 / 0.73 | 0.67 / 0.68 |
|  |  | 40 | 0.85 / 0.67 | 0.67 / 0.39 | 0.79 / 0.87 | 0.37 / 0.99 | 0.75 / 0.9 | 0.51 / 0.07 | 0.69 / 0.71 |
|  |  | 60 | 0.86 / 0.64 | 0.75 / 0.53 | 0.84 / 0.86 | 0.48 / 0.97 | 0.8 / 0.92 | 0.48 / 0.86 | 0.76 / 0.48 |
|  |  | 80 | 0.85 / 0.63 | 0.77 / 0.63 | 0.83 / 0.88 | 0.62 / 0.97 | 0.8 / 0.91 | 0.41 / 0.68 | 0.76 / 0.4 |


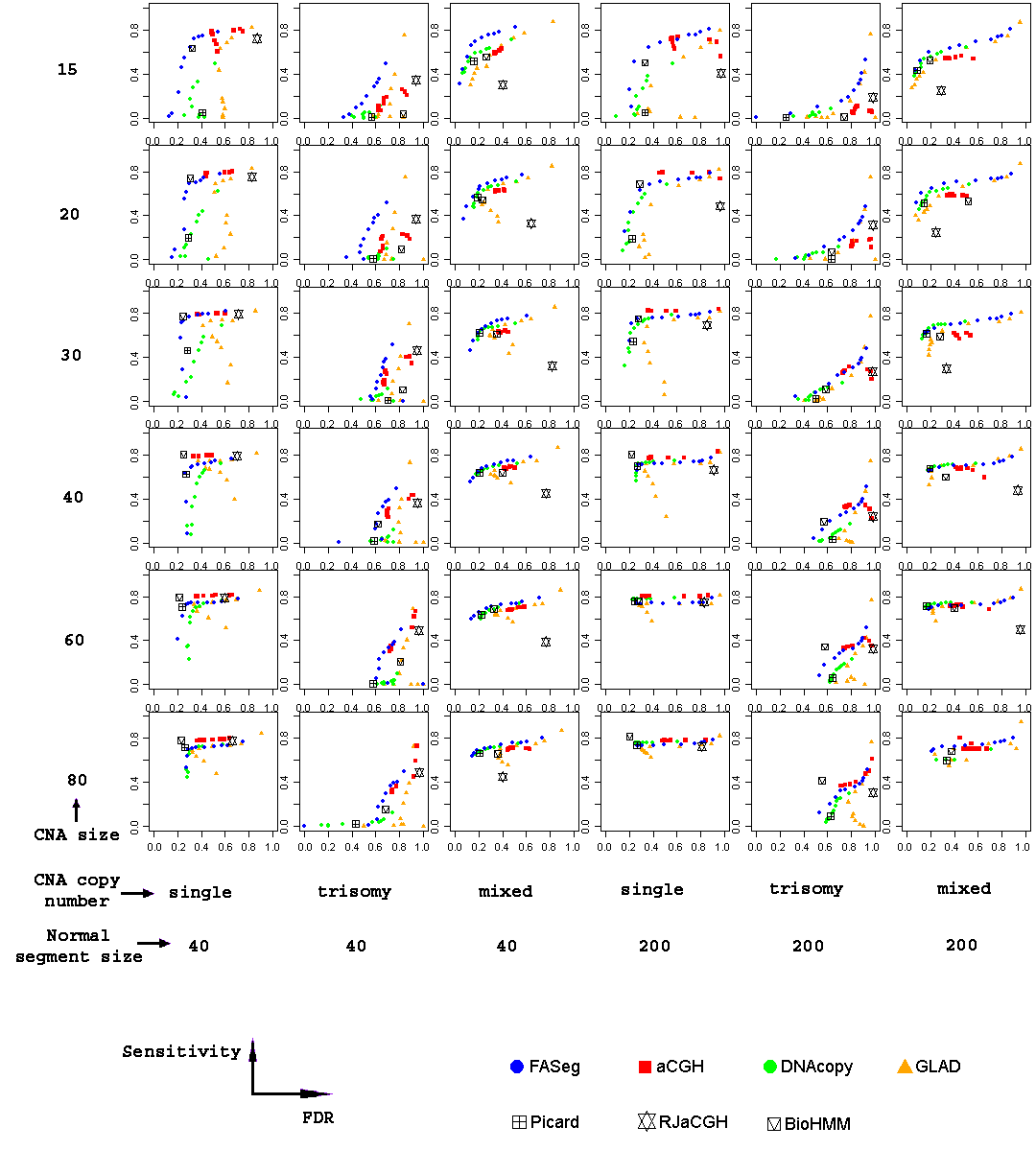


Supporting Figure 1. The comparison of the performance of seven methods with the tolerance distance set at 3.


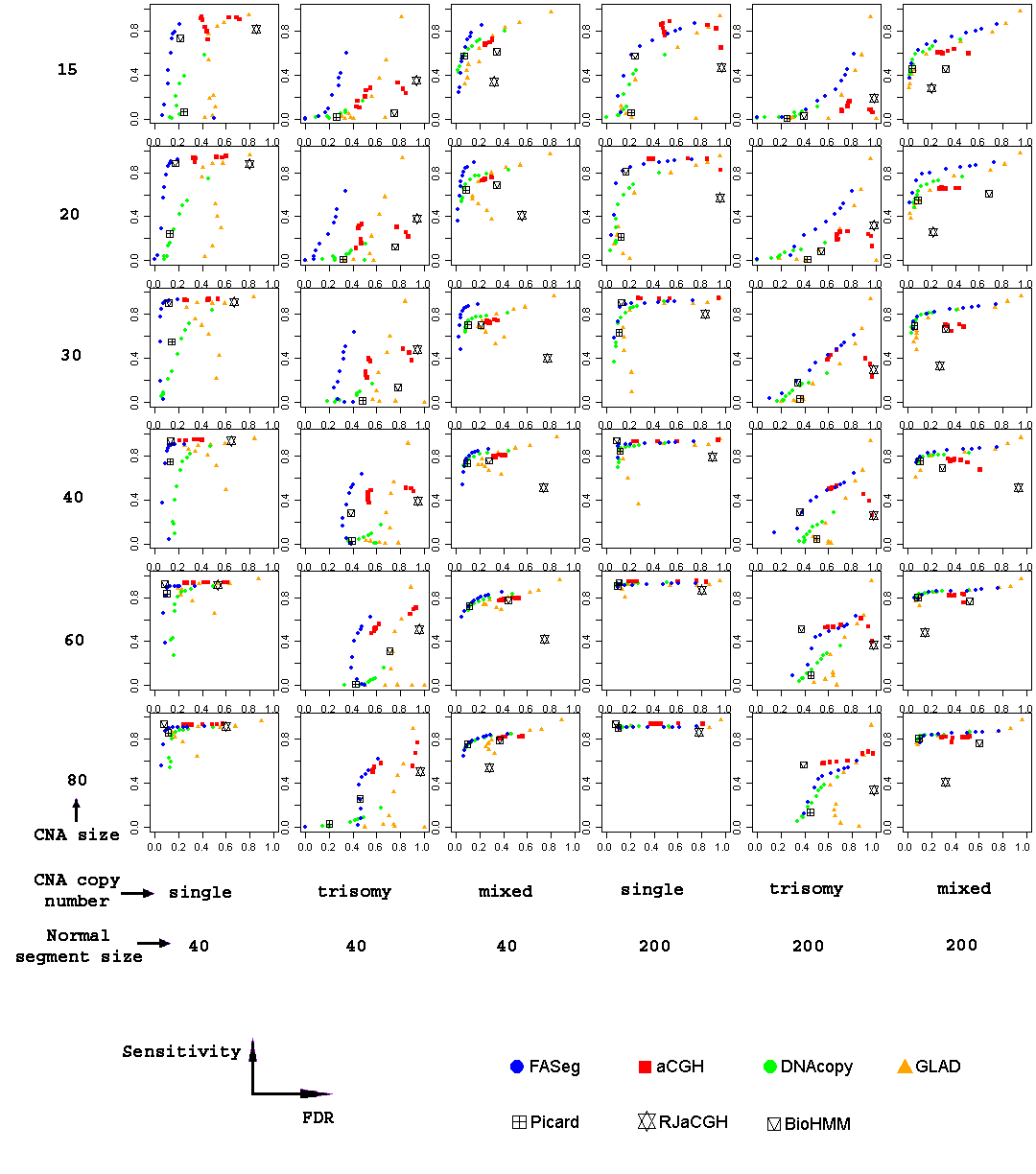


Supporting Figure 2. The comparison of the performance of seven methods with the tolerance distance set at 7.
